# Mitral Valve Prolapse Presenting as Unilateral Pulmonary Edema Mimicking Pneumonia

**DOI:** 10.1002/ccr3.73506

**Published:** 2026-09-11

**Authors:** Shigesato Inoue, Akihiro Jo, Hiroyuki Kumazoe, Kentaro Wakamatsu

**Affiliations:** ^1^ Department of Respiratory Medicine National Hospital Organization Omuta National Hospital Omuta Fukuoka Japan; ^2^ Department of Radiology National Hospital Organization Omuta National Hospital Omuta Fukuoka Japan

**Keywords:** acute mitral regurgitation, chordal rupture, mitral valve prolapse, transesophageal echocardiography, unilateral pulmonary edema

## Abstract

A 40‐year‐old man presented with right‐sided pulmonary infiltrates mimicking pneumonia. Imaging and transesophageal echocardiography revealed unilateral pulmonary edema (UPE) from acute severe mitral regurgitation (MR) due to chordal rupture. Unilateral infiltrates with rapid deterioration warrant early echocardiographic evaluation.

## Key Clinical Message

Unilateral pulmonary edema due to acute mitral regurgitation can closely mimic pneumonia. In patients with unilateral pulmonary infiltrates and rapid respiratory deterioration, particularly when the clinical course is atypical for infection, early echocardiographic evaluation should be considered to identify an underlying cardiac cause.

## 1 Introduction

Unilateral pulmonary edema (UPE) is an uncommon manifestation of cardiogenic pulmonary edema and is frequently misdiagnosed as pneumonia. Mitral regurgitation (MR) with an eccentric jet has been reported to cause right‐sided predominance [1]. We present a case of mitral valve prolapse mimicking pneumonia.

## 2 Case History

**FIGURE 1:**
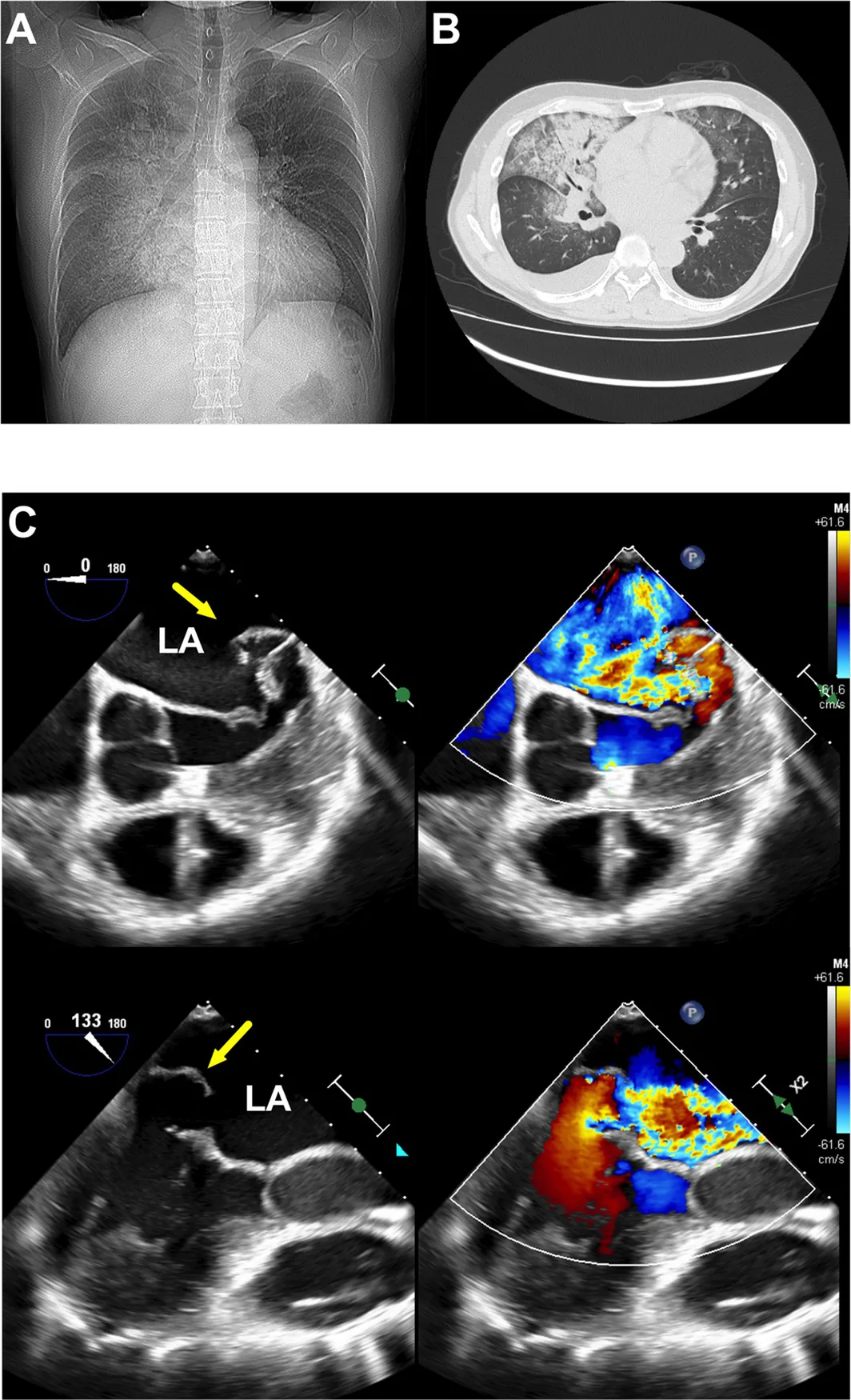
Mitral valve prolapse mimicking pneumonia on clinical imaging. (A) Chest radiography showed increased vascular markings with pulmonary infiltration. (B) Computed tomography demonstrated ground‐glass opacities with infiltrative changes and pleural effusion, predominantly in the right lung field. (C) Transesophageal echocardiography (Left) and Color Doppler images (Right) revealed mitral valve prolapse with eccentric mitral regurgitation, demonstrating a strong turbulent flow signal directed toward the left atrium. LA indicated the Left Atrium, and the yellow arrow showed the prolapsed mitral valve.

A previously healthy 40‐year‐old man presented with a 2‐day history of cough and rapidly progressive dyspnea. On admission, the patient was tachypneic (respiratory rate, 35/min) with hypoxemia (SpO_2_ 90% while receiving oxygen at 8 L/min via face mask). Blood pressure was 104/66 mmHg, and body temperature was 36.4°C. Electrocardiography showed sinus tachycardia (115 bpm) with left ventricular hypertrophy. Initial laboratory findings revealed a white blood cell count of 11,600/μL, C‐reactive protein of 0.58 mg/dL, and brain natriuretic peptide of 193 pg/mL. Chest radiography and computed tomography (Figure 1A,B) revealed unilateral pulmonary infiltrates predominantly in the right lung. Pneumonia was suspected, and antimicrobial therapy was initiated. Within 12 h after admission, respiratory failure rapidly progressed despite antimicrobial therapy. Oxygen demand increased to high‐flow nasal cannula (40 L/min, FiO_2_ 0.70). Because advanced cardiovascular and intensive care management was unavailable at our hospital, the patient was transferred by physician‐staffed helicopter to a tertiary referral center and required endotracheal intubation during transport. A systolic murmur was faintly audible; however, adequate auscultation was difficult due to tachypnea.

After transfer, transthoracic echocardiography suggested severe mitral regurgitation. Subsequent transesophageal echocardiography (Figure 1C) demonstrated prolapse of the posterior mitral leaflet with severe eccentric mitral regurgitation due to chordal rupture. The patient underwent urgent mitral valve repair 3 days after transfer. Intraoperative findings confirmed rupture of thick primary chordae tendineae attached to the P2 scallop, resulting in extensive prolapse of the P2 segment. Postoperatively, respiratory status improved rapidly, allowing extubation on postoperative Day 2. Renal function, which had been transiently impaired, gradually recovered thereafter. The patient was transferred for rehabilitation 30 days after admission, with complete resolution of the pulmonary infiltrates.

## 3 Discussion

This case illustrates that MR should be considered a culprit in the acute aggravation of unilateral pulmonary infiltrates. While cardiogenic pulmonary edema is typically bilateral, unilateral presentation occurs in approximately 2% of cases [2]. The mechanism of right‐sided UPE in MR involves an eccentric regurgitant jet directed toward the right pulmonary veins, particularly the right upper pulmonary vein. This jet is thought to increase local hydrostatic pressure and may cause flow reversal and localized edema. In our case, transesophageal echocardiography (Figure 1C) clearly demonstrated a potent, eccentric jet directed toward the right‐sided circulation, consistent with the predominant right‐sided infiltrates observed on radiography and CT (Figure 1A,B). Acute UPE caused by chordal rupture is frequently misdiagnosed as pneumonia due to its rapid onset, severe respiratory failure, and unilateral infiltrates [3]. In this patient, the absence of prior cardiac history, the near‐normal inflammatory markers, and the localized infiltrates initially favored an infectious etiology. A limitation of this report is that the proposed mechanism was inferred from echocardiographic findings rather than directly demonstrated physiologically.

This case underscores the necessity of considering acute MR in patients presenting with atypical unilateral infiltrates and rapid clinical deterioration. This educational message is particularly important for non‐cardiologists, including emergency and respiratory physicians, because unilateral pulmonary infiltrates are often initially attributed to pneumonia. Even when tachycardia or tachypnea masks cardiac murmurs, early echocardiographic evaluation—including transesophageal echocardiography when clinically indicated—is crucial for timely diagnosis and surgical intervention. Cardiogenic unilateral pulmonary edema should be considered in patients with unilateral infiltrates and rapid deterioration, even when pneumonia is initially suspected.

## Author Contributions

**Shigesato Inoue:** writing – review and editing, writing – original draft, conceptualization, investigation, visualization. **Akihiro Jo:** investigation. **Hiroyuki Kumazoe:** writing – review and editing, supervision, investigation, visualization. **Kentaro Wakamatsu:** writing – review and editing, supervision.

## Funding

The authors have nothing to report.

## Consent

The authors declare that written informed consent was obtained for the publication of this manuscript and its accompanying images and attest that the consent form used to obtain the patient's consent complies with the Journal's requirements as outlined in the author guidelines.

## Conflicts of Interest

The authors declare no conflicts of interest.

## Data Availability

The data that support the findings of this study are available from the corresponding author upon reasonable request.
